# The relationship between social support and Internet addiction among Chinese college freshmen: A mediated moderation model

**DOI:** 10.3389/fpsyg.2022.1031566

**Published:** 2023-01-04

**Authors:** Xiaoman Lu, Mengnan Zhang, Jingqiu Zhang

**Affiliations:** Department of Psychology, Shandong Normal University, Jinan, China

**Keywords:** social support, Internet addiction, network-related maladaptive cognition, gender difference, college freshmen

## Abstract

**Purpose:**

Internet addiction has become a worldwide mental health problem, and this problem is particularly prominent in China. Although current studies have shown that social support is closely related to Internet addiction, the mechanism of the relationship between the two is not clear at present. This study aimed to find out the influencing factors and the mechanism of Internet addiction among college freshmen, and to form scientific prevention and intervention plan on this basis.

**Method:**

This study adopts the cluster sampling method to select 322 college freshmen in a typical postsecondary school in Shandong Province, using Chinese Internet Addiction Scale (CIAS), Social Support Rating Scale (SSRS), and Network-related Maladaptive Cognition Scale (NRMCS) to investigate the relationship between social support, network-related maladaptive cognition, gender, and the degree of Internet addiction.

**Results:**

The findings of this study are as follows: (1) After controlling age and family location, social support had a significant negative predictive effect on Internet addiction; (2) Gender acted as a moderator between the relationship of social support and Internet addiction; and (3) Additionally, the moderating effect of gender was completely mediated by network-related maladaptive cognition.

**Conclusion:**

There is a mediated moderating effect between social support and Internet addiction. That is, gender plays a moderating role between social support and Internet addiction, and this moderating effect is mediated by network maladaptive cognition.

## Introduction

Internet addiction, also known as Internet dependence (Scherer and Bost, 1997), problematic Internet use (Shapira et al., 2000), pathological Internet use (Davis, 2001), compulsive Internet use (Meerkerk et al., 2006), and unregulated Internet usage (LaRose et al., 2003), is the “compulsive overuse of the Internet and irritable or emotional behavior when deprived of it” (Douglas et al., 2008). To better explain the characteristics of Internet addiction, some researchers have proposed the “physiological-psycho-social model” of Internet addiction (Lei et al., 2003; Griffiths, 2005; Malygin et al., 2017). In terms of the physiology aspect of this model, Internet addicts indulge on the Internet to maintain the resulting short-term and high-intensity excitement, which leads to a series of complex physiological and biochemical changes in the body over time, such as abnormal dopamine levels. Psychologically, Internet addicts repeatedly experience the excitement of using the Internet and the pain caused by stopping using it. As for social environmental factors, interpersonal indifference is one of the important causes of Internet addiction, which in turn aggravates the social separation of users.

Internet addiction has become a particularly prominent mental health problem in China (Shaw et al., 2008; Block, 2008; Mak et al., 2014). According to a survey conducted in China, the incidence of Internet addiction in the general population was 36.7%, and the incidence of severe Internet addiction was 2.8% (Li et al., 2021). More notably, as emerging adults, college students, especially freshmen, are still undergoing rapid changes in their brain neurobiology at the physiological level, which results in reinforcement events being experienced as particularly positive, and relatively weak associations with inhibitory structures (Agrawal et al., 2012; Sussman and Arnett, 2014). On a psychological level, young adults are in a stage of identity exploration and must complete a series of evolutionary tasks to successfully transition to adulthood (Arnett, 2014). In addition to the special physical and mental characteristics, young adults who wish to attend university are required to spend most of their time studying (Li, 2017). Once at university, students are prone to problematic Internet use behavior at their university campus, where they are free and independent, since they have less study pressure than at high school and unimpeded Internet access (Kuss, 2013). In conclusion, college freshmen are one of the most susceptible groups to Internet addiction (Kandell, 1998; Morgan and Cotten, 2003; Chou et al., 2005; Ni et al., 2009; Tsitsika et al., 2009). In one study, more than 80% of the 244 emerging adult college students had mild to moderate Internet addiction (Marzilli et al., 2020). In addition, a study on problematic Internet use in primary school, junior high school, senior high school, and college students using stratified sampling revealed significantly more problematic Internet use in college and senior high school students than in primary school and junior high school students, and significantly more problematic Internet use in junior high school students than in primary school students (Wang et al., 2020). That study also found more problematic Internet use in boys than in girls in primary and middle schools, but no significant sex-related difference in problematic Internet use in high school or university students. In addition, a study shows that the prevalence of Internet addiction among Chinese college freshmen is 17.4% (Yang et al., 2019). Several studies have shown that Internet addiction in college students is often accompanied by a series of interpersonal, educational, behavioral, physical, and mental health problems, including insomnia, anxiety, depression, and low self-esteem (Kittinger et al., 2012; Younes et al., 2016), which harm their academic and life development.

### The relationship between social support and internet addiction

The formation of Internet addiction is associated with many factors. It is generally believed that Internet addiction is the same as other addictive behaviors, whose formation is jointly affected by a person’s internal and external factors. Social support refers to the material and spiritual care or help from others during difficult times or emergencies, and a lack of social support is one external factor that can lead to Internet addiction (Sherbourne and Stewart, 1991; Xie and Kim, 2022). There is a close relationship between the level of social support and the degree of mental health of college students. Studies show that perceived family support and perceived teacher support significantly positively predict the life satisfaction of college students (Yalçın, 2011). Meanwhile, college students generally believe that they receive less support than they need, and this difference is associated with more severe depressive symptoms (Rankin et al., 2018). Two lines of research have demonstrated the influence of social support on Internet addiction. First, as a protective force of individuals in difficult times or emergencies, social support can meet an individual’s need of feeling belonging, love, and respect (Maslow, 1943, 1987). When there is a lack of adequate social support in real life, individuals may turn to the Internet to find dynamic social support groups to fulfill their own psychological needs; moreover, the virtuality and anonymity of the Internet provide a new and different way to establish interpersonal relationships (Suler, 1999; Wen, 2007; Esen, 2009; Zhang, 2021). According to the network use satisfaction theory, which was developed off the back of the media-based use satisfaction theory (Katz et al., 1973; Chou et al., 1999; Ruggiero, 2000), the Internet is essentially a mass media (Morris and Ogan, 1996), and various needs can be met through Internet use (McQuail, 1987). When the need for real-life social support in instead met online by using the Internet, this satisfaction will, in turn, stimulate individuals to use the Internet more frequently and for longer durations, which eventually leads to Internet dependence (Chou et al., 1999; Chou and Hsiao, 2000; Davis, 2001). Second, empirical studies have found that there is a correlation between social support and the degree of Internet addiction. For example, a study by Esen (2009) revealed that the degree of Internet addiction among adolescents decreases as support from parents and teachers increases. A cross-cultural study with Chinese international students (African and South Asian) showed that perceived social support is a predictor of Internet addiction among international students, and students from both cultures use the Internet excessively to compensate for unsatisfactory social support (Chaudhary, 2020). Another meta-analysis of 76 studies on Internet addiction in mainland China showed a moderate negative correlation between social support and Internet addiction (Lei et al., 2018). In addition, a cross-lag study among postsecondary first-year students by Zhang et al. (2018) found that social support negatively predicted Internet addiction, and the predictive relationship was unidirectional. Although all the above studies have shown that social support is closely associated with Internet addiction, the mechanism underlying this relationship is not yet clear. Therefore, this study explored what these mechanisms might be, based on previous results.

### The moderating role of gender

According to both the need satisfaction theory (Maslow, 1943, 1987; Suler, 1999; Taormina and Gao, 2013; Liu et al., 2016) and the Internet use satisfaction theory (Chou et al., 1999; Chou and Hsiao, 2000; Davis, 2001; Song et al., 2004), whether or not an individual lacking social support eventually forms Internet addiction largely depends on their motivation to seek social support, and whether they use the Internet as a way to meet their own psychological needs. Previous studies have found that men and women use the Internet for different reasons; namely, women attach more importance to the interpersonal relationship aspect of Internet use than do men, are more likely than men to seek and provide social support (Liebler and Sandefur, 2002), and tend to use the Internet as a tool to search for information and engage in interpersonal communication. However, men are more likely to use the Internet for entertainment or business purposes (Weiser, 2000; Lin et al., 2013). Therefore, we can speculate that gender influences the strength of the relationship between social support and Internet addiction; that is, social support has a greater predictive effect on women’s Internet addiction than on men’s. In other words, when women’s social support needs are not fully met in reality, they may be more likely than men to develop Internet addiction in the process of finding social support online. On the contrary, when women have sufficient social support in real life, they may no longer be addicted to the Internet. This prediction is also supported by the available evidence. For example, Block et al. (2014) found that women are more likely to develop social network addiction when they are depressed or have unmet social needs, and this is in large part because they see social networks as a way to fill a sense of emptiness. Using structural equation modeling, Mo et al. (2018) found that social support in women was more strongly correlated with emotional disorders, Internet use, and Internet addiction than it was in men. More importantly, a study on university students in Taiwan also supports the above conclusion (Yeh et al., 2008).

### The mediating role of network-related maladaptive cognition

In addition, many studies have shown that network-related maladaptive cognition can increase problematic Internet use behavior and lead to Internet addiction (Zhen et al., 2016; Zhou et al., 2019). Network-related maladaptive cognition refers to the phenomenon by which individuals have overly positive ideas and unrealistic expectations about the network world, and thus prefer it over the real world (Ding et al., 2018). Davis (2001) proposed a cognitive-behavioral model to explain the formation of Internet addiction, which holds that network-related maladaptive cognition is a key proximal factor that affects the formation, development, and maintenance of Internet addiction, and is the most stable predictor of Internet addiction. Meanwhile, the formation of network-related maladaptive cognition is influenced by remote factors (e.g., social support in the environment). This assertion can be explained by the need satisfaction theory; that is, when individuals experience relative deprivation or unsatisfied needs in real life, they are more likely to regard the online world as an effective way to meet their needs, which leads to network-related maladaptive cognition (Ding et al., 2018).

Based on the evidence presented thus far, it can be predicted that when individuals’ needs for belonging and love are not satisfied due to a lack of social support, they are more likely to use virtual networks as a way to compensate for these unmet psychological needs, which is exactly in line with the core characteristics of network-related maladaptive cognition. Therefore, we hypothesized that network-related maladaptive cognition can be predicted by social support, and may play a mediating role in the relationship between social support and Internet addiction. In addition, given that women pay more attention to interpersonal relationships and are more likely to seek social support than men, we predicted that women are more likely to experience a lack of social support in real life, and thus are more likely to exaggerate the positive role of the Internet and regard the Internet world as an efficient way to meet needs in the absence of social support. Previous studies have found that women are more likely to perceive social support on social networking sites than men (Tifferet, 2020), but it remains to be explored whether gender and social support can jointly affect perceptions of network-related maladaptive cognition and thus affect the severity of Internet addiction.

**Figure 1:**
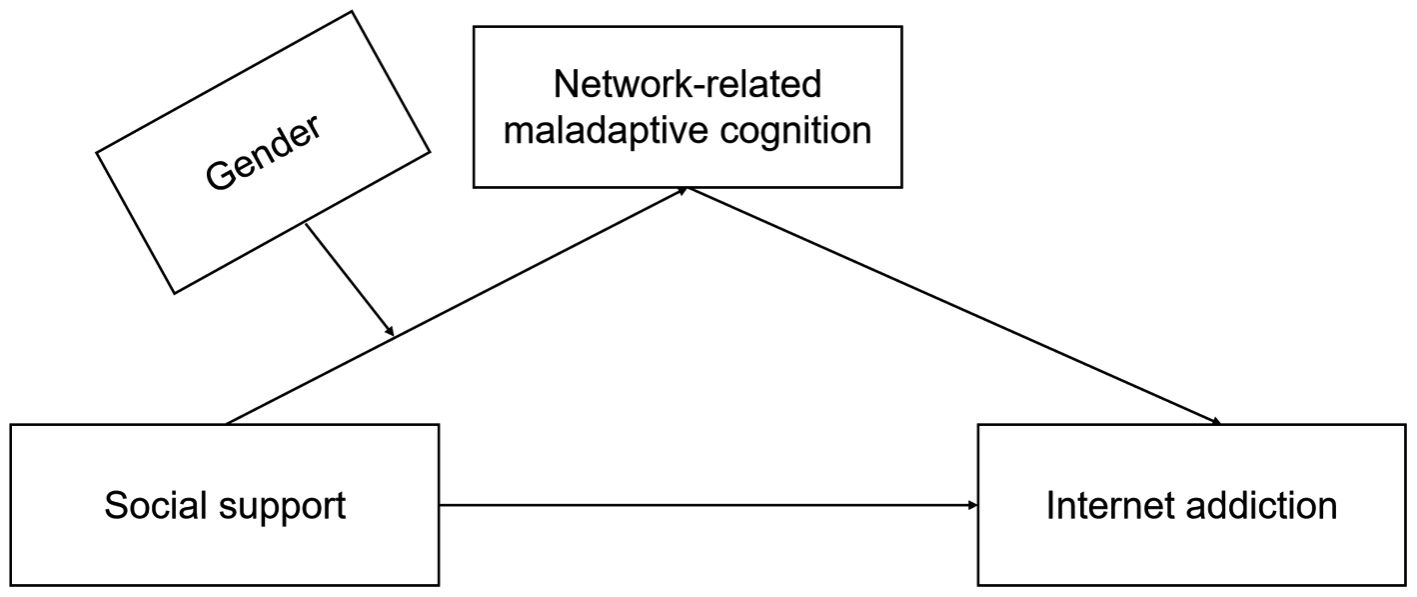
Conceptual model of research.

Based on the above discussion, it is particularly important to find out the influencing factors and mechanism of Internet addiction among college freshmen and to form scientific prevention and intervention programs on this basis. Therefore, this study further clarifies the relationship between social support and Internet addiction on the basis of previous studies and explores the specific influence mechanism of social support on Internet addiction by constructing and testing a mediated Moderation Model mediation regulation model (Figure 1). Firstly, this study explores the relationship between social support, gender, and Internet addiction, and whether gender plays a moderating role in the relationship between social support and Internet addiction. The second objective is to investigate whether network-related maladaptive cognition plays a mediating role between social support and Internet addiction, and whether or not the moderating effect of gender plays a role through network-related maladaptive cognition. We propose two hypotheses:

*H1*: Social support can significantly negatively predict the degree of Internet addiction, and gender plays a moderating role in the relationship between the two.

*H2*: Social support can not only directly and negatively predict Internet addiction, but also indirectly predict Internet addiction through network-related maladaptive cognition.

In other words, network-related maladaptive cognition plays a mediating role in the relationship between social support and Internet addiction. At the same time, the moderating effect of gender on the relationship between social support and Internet addiction can also indirectly affect the degree of Internet addiction through network-related maladaptive cognition.

## Materials and methods

### Procedures and participants

Based on a cross-sectional design, the current study used a non-probabilistic and cluster random sampling strategy among 6,221 Chinese college freshmen in a post-secondary school in Shandong Province. The inclusion criteria for participants in this study were: (1) participants were Chinese college freshmen at the beginning of the school year, (2) participants could use Chinese proficiently, and (3) participants consented voluntarily and were assured that the responses were confidential. With the permitting of the Shandong Normal University, a team of researchers, whose members were trained formally, was established to collect data through participants’ self-reporting.

In the first measurement, a total of 340 adolescents were enrolled. Overall, 322 students whose mean age is 18.31 years (*SD* = 0.85) responded to the questionnaire (the response rate was 94.71% and sampling ratio is 5.47%). comprising 144 males (44.72%) and 178 females (55.28%). There were 182 (56.52%) students living in the city or metropolitan 135 (41.93%) students living in the village or town and five students did not report (1.55%). Of the initial sample, 18 students provided invalid responses (e.g., the participants responded to the scale regularly on purpose). The study was conducted according to the guidelines of the Declaration of Helsinki and approved by the Institutional Review Board (or Ethics Committee) of the Ethics Committee of Shandong Normal University (protocol code sdnu-2020121523). Besides, written informed consent was obtained from all individual participants in this study. At the same time, for those participants who were minors in China, written informed consent from their parents was also obtained.

### Measurements

#### The Chinese Internet addiction scale

The Chinese Internet Addiction Scale (CIAS) developed by Chen et al. (2003) was employed in the current study to measure Internet addiction. It included 19 items with four dimensions: first, compulsive Internet use and withdrawal from Internet addiction (six items, e.g., “Whenever I’m not online for a while, I feel like I’m missing something”); second, tolerance of Internet addiction (four items, e.g., “I find myself spending more and more time online”); third, time management problems (five items, e.g., “I found myself investing more on the Internet than interacting with my friends”), and fourth, interpersonal and health problems (four items, e.g., “More than once, I have not slept 4 h because of the Internet”). A four-point scale (1 = complete inconformity, 2 = comparatively inconformity, 3 = comparatively conformity, and 4 = complete conformity) was used for the response. The higher the score in each subscale, the higher level of each dimension is. In the end, the responses were summed across all items, with higher scores representing greater Internet addiction. To ensure the reliability and validity of the scale, reliability analysis (Cronbach’s alpha) was applied to the responses for this sample. Good reliability was shown with Cronbach’s alpha value of 0.91 for the total scale and Cronbach’s alpha values of 0.77, 0.73, 0.82, and 0.72 for the four subscales. At the same time, the confirmatory factor analysis indicators of the questionnaire are *x*^2^/df = 15.084, RMSEA = 0.099, TLI = 0.779, CFI = 0.811.

#### The social support rating scale

The Social Support Rating Scale (SSRS) compiled by Xiao (1994) was adopted in the current study to measure social support. It included 10 items with three dimensions: first, objective support (three items, e.g., “What are some of the sources of financial support and practical help you have received in the past in times of emergency”); second, subjective support (four items, e.g., “How many close friends do you have that you can count on for support and help?”); third, support utilization [three items; e.g., “How do you talk about your troubles? (Select only one item)”]. There are four corresponding options after each item for the response. The higher the score in each subscale, the higher level of each dimension is. In the end, the responses were summed across all items, with higher scores representing more social support. To ensure the reliability and validity of the scale, reliability analysis (Cronbach’s alpha) was applied to the responses for this study. Good reliability was shown with Cronbach’s alpha value of 0.66 for the scale and Cronbach’s alpha values of 0.51, 0.49, and 0.51 for the three subscales. At the same time, the confirmatory factor analysis indicators of the questionnaire are *x*^2^/df = 7.192, RMSEA = 0.082, TLI = 0.449, CFI = 0.534.

#### Network-related maladaptive cognition scale

Network-related maladaptive cognition was measured by the Network-related Maladaptive Cognition Scale (NRMCS), which was adapted by Liang (2000) from the Online Cognition Scale developed by Davis et al. (2002) according to the cognitive-behavioral model (Davis, 2001). The scale consists of 14 items with three dimensions: first, Internet comfort (five items; e.g., “I get more respect online than I do in real life”); second, weak impulse control (five items; e.g., “I often experience a “rush” or heightened mood when I’m online”); and third, escape and retreat (four items; e.g., “I do not need to think about real problems when I’m online”). Students rated each item on a five-point scale, ranging from 1 (complete inconformity) to 5 (complete conformity). The responses were summed across all items, with higher scores representing greater network-related maladaptive cognition. To ensure the reliability and validity of the scale, reliability analysis (Cronbach’s alpha) was applied to the responses for this sample. Good reliability was shown with Cronbach’s alpha value of 0.87 for the scale and Cronbach’s alpha values of 0.81, 0.81, and 0.70 for the three subscales. At the same time, the confirmatory factor analysis indicators of the questionnaire are *x*^2^/df = 2.661, RMSEA = 0.072, TLI = 0.901, CFI = 0.920.

### Data processing

Statistical Package for Social Sciences (SPSS) 26.0 (Shandong Normal University, Jinan, China) was used to input data and carry out the descriptive analysis, correlation analysis, multiple regression, and simple slope testing after missing data were analyzed through the estimation option. Specifically, Pearson correlation analysis was used to assess the relationships between the key variables. After that, the mediated-moderation effect was investigated first by multiple regression and then further by simple slope analysis. What is more, the analysis of the robustness of the mediation effect using the bootstrap estimation process with 2,000 replications was carried out by Mplus 8.3 (Shandong Normal University, Jinan, China) software. All values of *p* were two-tailed, and a value of *p* of <0.05 was considered statistically significant.

## Results

### Common method bias detection

The research data were collected by self-report questionnaires, which may have led to common method bias (CMB). As ignoring CMB will threaten its validity, in the process of data collection, CMB was controlled by an anonymous questionnaire and other methods. After the data collection, Harman’s single factor test was conducted on the variables reported by parents (Podsakoff et al., 2003). The results showed that there are 13 factors whose characteristic root was greater than 1, and the interpretation rate of the first factor was 24.81%, which was less than the critical standard of 40%, indicating that there was no obvious CMB in this study.

### Correlation analysis of main variables

The means and standard deviations of the variables are shown in Table 1, as well as the correlations between variables. According to Table 1, social support was negatively correlated with network-related maladaptive cognition (*r* = −0.24, *p* < 0.001), Internet addiction (*r* = −0.21, *p* < 0.001), while network-related maladaptive cognition was positively correlated with Internet addiction (*r* = 0.76, *p* < 0.001). At the same time, gender was negatively correlated with social support (*r* = −0.14, *p* < 0.05) and positively correlated with age (*r* = 0.18, *p* < 0.01) as well as network-related maladaptive cognition (*r* = −0.20, *p* < 0.001), while the correlation between gender and other variables is not significant. Besides, home location was positively correlated with age (*r* = 0.23, *p* < 0.001).

**Table 1:** Means, standard deviation, and correlations (*n* = 322).

Variables	*M*	*SD*	1	2	3	4	5	6
1. Age	18.31	0.85	1					
2. Home location^a^	0.57	0.50	0.23^***^	1				
3. Social support	40.74	5.20	−0.02	−0.01	1			
4. Gender^b^	0.45	0.50	0.18^**^	0.06	−0.14^*^	1		
5. NMC	33.16	10.17	−0.05	−0.07	−0.24^***^	0.20^***^	1	
6. Internet addiction	34.32	9.78	0.04	0.03	−0.21^***^	0.10	0.76^***^	1

Home location^a^ is the dummy variable, Rural = 1, City = 0. Gender^b^ is the dummy variable, Female = 0, Male = 1. ^*^p < 0.05, ^**^p < 0.01, ^***^p < 0.001. NMC, network-related maladaptive cognition.

### Mediated-moderation effect analysis

To investigate the moderation effect of gender between social support and network-related maladaptive cognition and the mediation effect of network-related maladaptive cognition between social support and Internet addiction, this study referred to the work of Ye and Wen (2013) to verify the mediated-moderation model by testing three regression equations. Except for gender and home location, all variables used in this study were processed by standardized and the product term (social support × gender) was calculated, with sociodemographic variables including age and home location being controlled. Multiple regression analysis results are shown in Table 2.

**Table 2:** A regression test of the mediated moderating effect (*n* = 322).

Variable	Step 1 IA			Step 2 NMC			Step 3 IA		
	*B*	*SE*	*t*	*B*	*SE*	*t*	*B*	*SE*	*t*
Age	−0.03	0.07	−0.41	−0.16	0.07	−2.21^*^	0.09	0.05	1.75
Home location^a^	0.06	0.12	0.55	−0.08	0.11	−0.72	0.13	0.08	1.68
Social support	−0.45	0.09	−4.9^***^	−0.41	0.09	−4.62^***^	−0.12	0.07	−1.80
Gender^b^	0.19	0.12	1.62	0.45	0.11	4.02^***^	−0.14	0.08	−1.73
Social support × gender	0.41	0.12	3.45^**^	0.31	0.11	2.76^**^	0.15	0.08	1.82
NMC							0.76	0.04	18.27^***^
*R* ^2^	0.09			0.14			0.58		
*F*	5.86^***^			9.57^***^			66.23^***^		

Home location^a^ is the dummy variable, Rural = 1, City = 0. Gender^b^ is the dummy variable, Female = 0, Male = 1. ^*^p < 0.05, ^**^p < 0.01, ^***^p < 0.001. IA, internet addiction; NMC, network-related maladaptive cognition.

**Figure 2:**
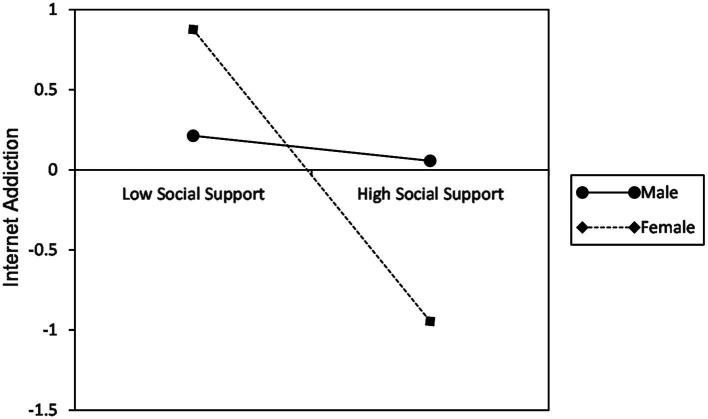
Moderating effect of gender on social support and Internet addiction.

In the first step, we calculated the regression of Internet addiction to social support and gender (the moderator) and social support × gender. The results showed that the regression coefficient of social support × gender was significant (B = 0.41, *p* < 0.01), which means that gender plays a significant role in moderating the relationship between social support and Internet addiction. According to simple slope testing (Figure 2), for females, Internet addiction was significantly negatively predicted by social support (B = −0.46, *p* < 0.001). In other words, females’ Internet addiction has a dramatic growth as the social support goes down. However, this effect disappeared in males (B = −0.04, *p* = 0.587).

**Figure 3:**
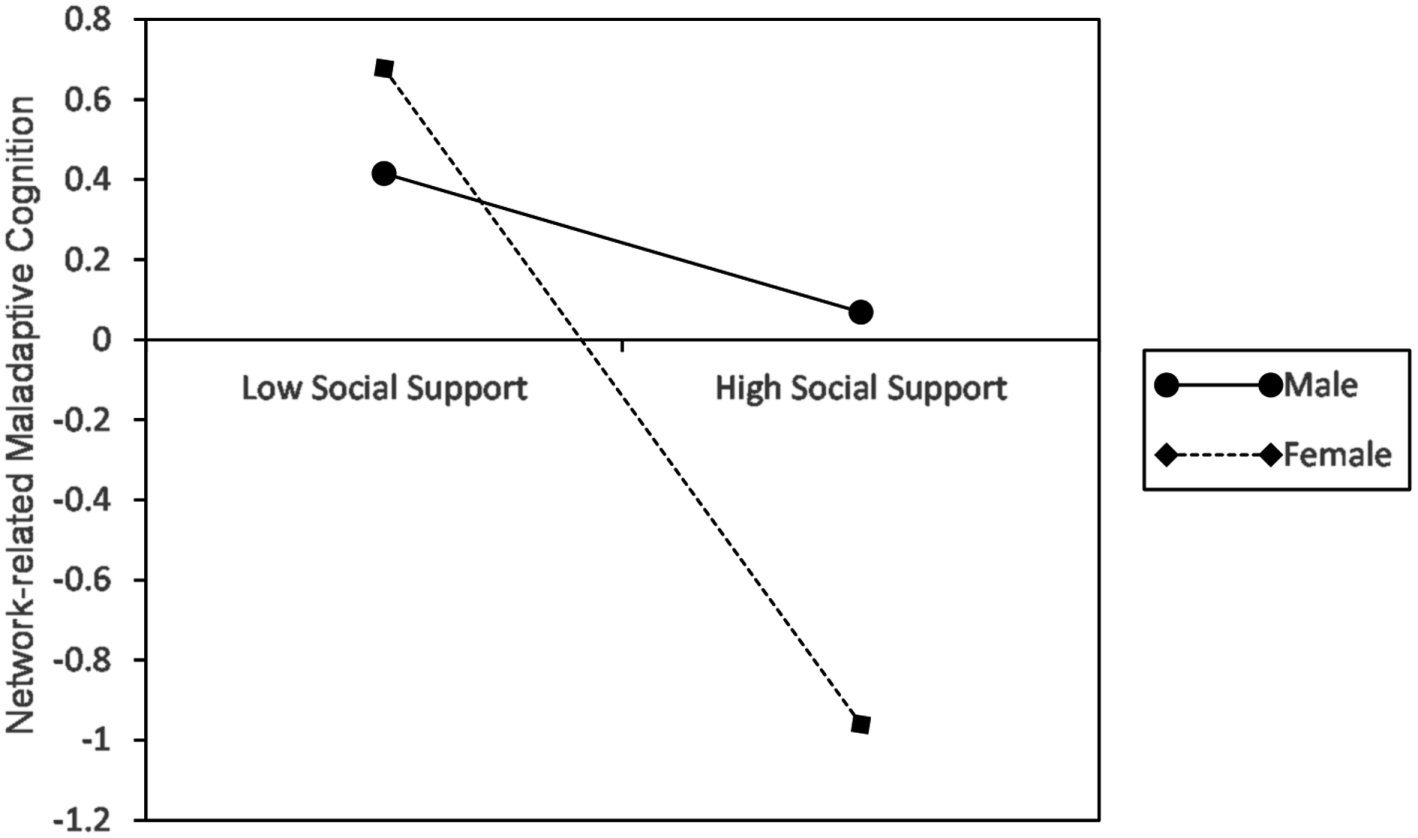
Moderating effect of gender on social support and network-related maladaptive cognition.

In the second step, we calculated the regression of network-related maladaptive cognition to social support; gender and social support × gender. The results showed that the coefficient of social support × gender was also significant (B = 0.31, *p* < 0.01), which means that gender plays a significant role in moderating the relationship between social support and network-related maladaptive cognition. According to simple slope testing (Figure 3), for females, network-related maladaptive cognition was significantly negatively predicted by social support (B = −0.41, *p* < 0.001). In other words, females’ network-related maladaptive cognition has a sharp drop as the social support increases. However, this effect disappeared in males (B = −0.09, *p* = 0.167).

Lastly, in the third step, we calculated the regression of Internet addiction to social support and gender; network-related maladaptive cognition and social support × gender. It showed that the regression coefficient of the product term to Internet addiction was not significant, while the regression coefficient of network-related maladaptive cognition was still significant (B = 0.76, *p* < 0.001), which indicates that network-related maladaptive cognition totally mediates the moderation of gender on social support and Internet addiction.

Further, we use the bootstrap estimation process to study the robustness of this mediation effect. The results showed that 95% confidence interval was significantly correlated with the mediation effect. Social support significantly and indirectly influenced Internet addiction through network-related maladaptive cognition, the indirect effect is estimated to be −0.31(95% confidence interval [−0.45 – −0.17]), and the interaction between social support and gender also influenced Internet addiction significantly through network-related maladaptive cognition, the indirect effect is estimated to be 0.24 (95% confidence interval [0.06–0.43]).

## Discussion

### The relationship between social support and Internet addiction

Using correlation and regression analyses, we found that social support negatively predicted the degree of Internet addiction. That is, less social support was associated with a greater severity of addiction. This result is consistent with those of multiple previous empirical studies (Esen, 2009; Zhang et al., 2018; Cui and Chi, 2021), and supports evidence that social support is an important predictor of Internet addiction, and that a lack of social support tends to lead to Internet addiction. The main effect model of social support (Liu et al., 2017) shows that the care, help, and support received by individuals in difficult or emergency situations can meet the needs of belonging, love, and respect, eliminate pressure, and tension, and contribute to physical and mental health (Cohen and Syme, 1985). For college freshmen in particular, the unique stage of psychological development and new learning and living environments come with all kinds of pressures and difficulties, such that they need more support from family, peers, and teachers to protect them from the influence of risk factors in the environment (Yildirim, 2004). According to the compensation model proposed by Kardefelt-Winther (2014), psychosocial problems or unmet real-life needs generate motivation to use the Internet; thus, if college freshmen’s social support needs are not met in real life, they are likely to search for social support to satisfy their psychological needs in the virtual and anonymous online world. However, according to the network use satisfaction theory (Katz et al., 1973; Chou et al., 1999; Ruggiero, 2000), the Internet is essentially a mass media (Morris and Ogan, 1996), and various needs can be met through Internet use (McQuail, 1987). As a result, once psychological needs are satisfied by using the Internet, such satisfaction leads to more frequent and longer duration Internet use, which ultimately leads to addiction (Chou et al., 1999; Chou and Hsiao, 2000; Davis, 2001; Song et al., 2004). Overall, the present results clearly demonstrate that a lack of social support is an important external factor that leads to the formation of Internet addiction.

### The moderating effect of gender

We found a significant correlation between gender and social support, whereby women had more social support than men, which is consistent with the results reported by Tamres et al. (2002) and Denton et al. (2004). After controlling for family location and age, we found that gender moderated the relationship between social support and Internet addiction. Specifically, social support significantly negatively predicted the degree of Internet addiction of female college freshmen, but not of male college freshmen. This result is consistent with H1 and supports previous findings that women are more likely to suffer from social network addiction than men when their social needs are not fully met (Block et al., 2014; Mo et al., 2018). According to network use satisfaction theory, the repeated reinforcement of satisfaction obtained in the network world is a key to making individuals addicted to the Internet. Therefore, when individuals lack social support in the real world, the strength of their motivation to seek social support and whether they use the Internet as a tool to compensate for psychological needs can, to a large extent, predict whether that individual will develop Internet addiction. It can be speculated that the moderating effect of gender in the relationship between social support and Internet addiction found in this study can be explained by the difference in the motivation of male and female Internet users. That is, women attach more importance to interpersonal relationships than men and have stronger motivation to seek social support (Liebler and Sandefur, 2002), and at the same time, they tend to use the Internet as a tool for searching information and engaging in interpersonal communication (Weiser, 2000; Lin et al., 2013). Therefore, a woman who is lacking social support is more likely to turn to the Internet, which she perceives as a reliable source of social support, and in turn gain a strong sense of compensation. In contrast, men rarely make up for their lack of social support online, both because they are less motivated to seek social support than women, and because they use the Internet less as a way of seeking information and engaging in interpersonal communication. This could also explain why the correlation coefficient between gender and Internet addiction was not significant in this study; that is, when women have the same high degree of social support as men, the degree of Internet addiction is very low, but when they have the same low degree of social support as men, the degree of Internet addiction is very high.

### The mediating effect of network-related maladaptive cognition

We found that social support not only directly affects Internet addiction, but also indirectly affects Internet addiction through network-related maladaptive cognition, which is the mediating variable. This result is supported by the need satisfaction theory and cognitive-behavioral model, which follows the model of “reality discomfort → network-related maladaptive cognition → addictive behavior” (Ding et al., 2018). Specifically, according to the need satisfaction theory, when individuals lacking social support in real life get their needs for social support satisfied by using the Internet, this satisfaction will in turn stimulate them to regard the online world as an effective way to meet their needs, thus leading to the occurrence of network maladaptive cognition. According to the cognitive-model, network maladaptive cognition is a key proximal factor affecting the formation, development and maintenance of Internet addiction (Davis, 2001; Song et al., 2004). Therefore, the increase of individual network maladaptive cognition promotes the formation of individual Internet addiction. In addition, the moderating effect of gender on the relationship between social support and Internet addiction was entirely mediated by network-related maladaptive cognition. Specifically, when women had sufficient social support, they had few inappropriate expectations of the Internet compared with men. Therefore, in this study, the correlation coefficient between gender and network-related maladaptive cognition was still significant, even though Internet maladaptive cognition of women increases with the decrease in social support. However, when there was a lack of social support, women were more likely to obtain social support through the Internet; this is because the lack of social support in women, who are more eager for social support, causes overly positive ideas about the Internet and inappropriate expectations, such as being more likely to perceive Internet use as an effective way to meet social support needs. This result indicates that network-related maladaptive cognition is a key mediating variable. As an important variable of individual cognition, it is not only affected by external environmental resources and individual physical and mental characteristics, but can also effectively predict an individual’s own Internet addiction severity.

### The application value of this study

Based on relevant theories, this study constructed a mediation model to reveal the relationships among social support, network-related maladaptive cognition, gender, and Internet addiction. These findings are of great significance for the formation of intervention programs for Internet addiction among freshmen. Firstly, this study found that social support is an important predictor of Internet addiction among freshmen, and lack of social support often leads to their Internet addiction. Therefore, in the actual prevention and intervention work, care and help should be given to students, and attention should be paid to the cultivation of students’ interpersonal relationships and communication skills so that they can get enough social support in real life. Secondly, in practical work, attention should be paid to the different effects of social support on Internet addiction among students of different genders, so as to facilitate the discovery of the main causes of Internet addiction and provide effective intervention and treatment measures. For example, when girls suffer from Internet addiction, according to the results of this study, educators can consider more whether it is because of the problem of girls’ social support and whether it is necessary to supplement them with social support from real life. For example, educators can give them more care and help them build friendships and better relationships with their families. At the same time, since this study found that network-related maladaptive cognition is a proximal factor of forming Internet addiction, more attention should be paid to educating and cultivating students at the cognitive level to eliminate their unreasonable beliefs about the Internet. Finally, as freshmen, students should actively seek and establish real-life sources of social support, such as participating in more college associations, attending more class gatherings, and communicating with their parents on the phone. In addition, college students should consciously correct their inappropriate expectations on the Internet so that they can better adapt to the learning environment in real life and have a healthy body and mind.

### Limitations and suggestions for future studies

Although this research has achieved certain results, there are still some shortcomings. First, this study is a cross-sectional study, so it cannot prove the causal relationship between variables. Further follow-up studies are needed to test the conclusions of this study in the future. Second, the subjects selected for this study are freshmen from a university in Shandong. They are in a special stage of psychological development and a new learning and living environment. Therefore, the conclusions of the study should be extended to other groups with caution and can be further tested in other groups in the future. Thirdly, based on the bio-psycho-social model (Griffiths, 2005) Internet addiction is the result of the interaction between individual genotypes, psychological characteristics, and social environment. In this study, we explored the influence of social support, a social-environmental factor, on the formation of Internet addiction, and considered the mediating role of network-related maladaptive cognition, a cognitive variable, according to the cognitive-behavioral model. However, emotional/behavioral functions and emotional regulation are also closely related to the formation of Internet addiction, and young people may have a higher risk of Internet addiction when trying to cope with psychological problems (Yan et al., 2014). For example, emerging adults may attempt to control anticipatory anxiety related to ascetic stressful situations and life events through excessive use of the Internet (Lin et al., 2020); Emerging adults with depression use the Internet as a strategy to escape and cope with feelings of sadness and low self-esteem (Young, 2017). Previous studies have shown that the psychopathological symptoms of emerging adults mediate the relationship between environmental risk factors and Internet addiction (Jang et al., 2012; Li et al., 2016), however, this study did not consider the content of emotions, and further studies can be carried out in the future.

## Conclusion

In summary, the current study aimed to identify the influencing factors and the mechanism of Internet addiction among college freshmen. As expected, our findings indicate that: (1) After controlling age and family location, social support had a significant negative predictive effect on Internet addiction; (2) Gender acted as a moderator between the relationship of social support and Internet addiction; and (3) Additionally, the moderating effect of gender was completely mediated by network-related maladaptive cognition.

In addition, there is a mediated moderating effect between social support and Internet addiction, that is, gender plays a moderating role between social support and Internet addiction, and this moderating effect is mediated by network-related maladaptive cognition.

## Data availability statement

The data that support the findings of this study are available from the corresponding author. Requests to access the datasets should be directed to the corresponding author.

## Ethics statement

The study was conducted according to the guidelines of the Declaration of Helsinki and approved by the Institutional Review Board of the Ethics Committee of Shandong Normal University (protocol code sdnu-2020121523). Written informed consent to participate in this study was provided by the participants themselves or, for minors, their legal guardians/next of kin.

## Author contributions

XL, MZ, and JZ contributed to the study conception and design, and performed material preparation and data collection and analysis. The first draft of the manuscript was written by XL and MZ. JZ commented on previous versions of the manuscript. All authors contributed to the article and approved the submitted version.

## Funding

This research was funded by the National Students Innovation and Entrepreneurship Training Program of Shandong Normal University, China (grant number 202110445103), the National Students Innovation and Entrepreneurship Training Program of Shandong Normal University, China (grant number 2022210445004X), the National Students Innovation and Entrepreneurship Training Program of Shandong Normal University, China (grant number 202210445001), and the National Students Innovation and Entrepreneurship Training Program of Shandong Normal University, China (grant number 2021180124).

## Conflict of interest

The authors declare that the research was conducted in the absence of any commercial or financial relationships that could be construed as a potential conflict of interest.

## Publisher’s note

All claims expressed in this article are solely those of the authors and do not necessarily represent those of their affiliated organizations, or those of the publisher, the editors and the reviewers. Any product that may be evaluated in this article, or claim that may be made by its manufacturer, is not guaranteed or endorsed by the publisher.
