# Erratum: The relationship between social support and Internet addiction among Chinese college freshmen: A mediated moderation model

**DOI:** 10.3389/fpsyg.2023.1156399

**Published:** 2023-02-10

**Authors:** Frontiers Production Office

**Affiliations:** Frontiers Media SA, Lausanne, Switzerland

**Keywords:** social support, Internet addiction, network-related maladaptive cognition, gender difference, college freshmen

Due to a production error, the DOI for this article was incorrectly registered as 10.3389/fpsyg.2023.1031566. The correct DOI for the article is 10.3389/fpsyg.2022.1031566.

The publisher apologizes for this mistake. The original version of this article has been updated.

